# Mitochondrion-associated protein LRPPRC suppresses the initiation of basal levels of autophagy via enhancing Bcl-2 stability

**DOI:** 10.1042/BJ20130306

**Published:** 2013-08-29

**Authors:** Jing Zou, Fei Yue, Xianhan Jiang, Wenjiao Li, Jinglin Yi, Leyuan Liu

**Affiliations:** *Medical College, Nanchang University, No. 461 Bayi Road, Nanchang, Jiangxi Province 330006, China; †Center for Cancer and Stem Cell Biology, Institute of Biosciences and Technology, Texas A&M Health Science Center, 2121 W. Holcombe Blvd, Houston, TX, 77030, U.S.A.

**Keywords:** ATG5, autophagy, Beclin 1, class III phosphoinositide 3-kinase (PI3KCIII), leucine-rich pentatricopeptide repeat-containing (LRPPRC), microtubule-associated protein 1 small form (MAP1S), mitochondrion, p27, AMPK, AMP-activated protein kinase, eIF4E, eukaryotic initiation factor 4E, HEK, human embryonic kidney, HRP, horseradish peroxidase, LAMP, lysosome-associated membrane protein, LC3, light chain 3, LRPPRC, leucine-rich pentatricopeptide repeat-containing, LSFC, Leigh syndrome, French-Canadian type, MAP1, microtubule-associated protein 1, MAP1S, MAP1 small form, mTOR, mammalian target of rapamycin, PI3K, phosphoinositide 3-kinase, PI3KCIII, class III PI3K, Tom20, translocase of the mitochondrial outer membrane 20, Vps, vacuolar protein sorting

## Abstract

The mitochondrion-associated protein LRPPRC (leucine-rich pentatricopeptide repeat-containing) interacts with one of the microtubule-associated protein family members MAP1S (microtubule-associated protein 1 small form), originally named C19ORF5 (chromosome 19 open reading frame 5), to form a complex. MAP1S interacts with LC3 (light chain 3), the mammalian homologue of yeast autophagy marker ATG8 and one of the most important autophagy markers in mammalian cells, and helps the attachment of autophagosomes with microtubules for trafficking and recruitment of substrate mitochondria into autophagosomes for degradation. MAP1S activates autophagosomal biogenesis and degradation to remove misfolded/aggregated proteins and dysfunctional organelles such as mitochondria and suppress oxidative stress-induced genomic instability and tumorigenesis. Previously, various studies have attributed LRPPRC nucleic acid-associated functions. Instead, in the present study, we show that LRPPRC associates with mitochondria, interacts with Beclin 1 and Bcl-2 and forms a ternary complex to maintain the stability of Bcl-2. Suppression of LRPPRC leads to reduction in mitochondrial potential and reduction in Bcl-2. Lower levels of Bcl-2 lead to release of more Beclin 1 to form the Beclin 1–PI3KCIII (class III phosphoinositide 3-kinase) complex to activate autophagy and accelerate the turnover of dysfunctional mitochondria through the PI3K (phosphoinositide 3-kinase)/Akt/mTOR (mammalian target of rapamycin) pathway. The activation of autophagy induced by LRPPRC suppression occurs upstream of the ATG5–ATG12 conjugate-mediated conversion of LC3-I into LC3-II and has been confirmed in multiple mammalian cell lines with multiple autophagy markers including the size of GFP–LC3 punctate foci, the intensity of LC3-II and p62 protein and the size of the vacuolar structure. The activated autophagy enhances the removal of mitochondria through lysosomes. LRPPRC therefore acts to suppress the initiation of basal levels of autophagy to clean up dysfunctional mitochondria and other cellular debris during the normal cell cycle.

## Abstract 2

Mitochondrial protein LRPPRC interacts with LC3-interactive microtubule-associated MAP1S and regulates autophagy. It interacts with Beclin 1 and Bcl-2 to form a ternary complex to maintain Bcl-2 stability. LRPPRC suppression enriches the Beclin 1-PI3KCIII complex to activate autophagy and mitophagy.

## INTRODUCTION

Autophagy, or self-digestion, is a process that begins with the formation of isolation membranes [1]. The isolation membrane engulfs substrates including dysfunctional organelles, misfolded/aggregated proteins and/or other macromolecules to form autophagosomes which migrate along acetylated microtubules to fuse with lysosomes to generate autolysosomes in which substrates are degraded [1–4]. The mitochondrion is one of the most prominent and vital types of organelle in eukaryotic cells. During cell cycling, mitochondria are constantly synthesized, used, damaged and removed through autophagy (hereinafter referred to as mitophagy) [5,6]. In mammalian cells, autophagy initiation is regulated either by growth factors through the PI3K (phosphoinositide 3-kinase)/Akt/mTOR (mammalian target of rapamycin) pathway [7] or by nutrients through the LKB1/AMPK (AMP-activated protein kinase)/mTOR pathway [8,9]. The anti-apoptotic proteins of the Bcl-2 family exhibit opposite effects on autophagy initiation through the two different pathways. Bcl-2 sequesters Beclin 1 and prevents its association with PI3KCIII (class III PI3K) to suppress autophagy initiation through the PI3K/Akt/mTOR pathway [7,10] or enhances levels of p27 to activate autophagy through the LKB1/AMPK/mTOR pathway [8,9]. The precursor of LC3 (light chain 3), a homologue of the yeast autophagy marker ATG8 and an interactive protein of the MAP1 (microtubule-associated protein 1) family [11–13], is truncated to form the cytosolic LC3-I and conjugated further with phosphatidylethanolamine to create the isolation-membrane-associated LC3-II with the assistance of ATG5–ATG12 conjugates [14,15]. Dysfunctional mitochondria are separated from the healthy ones and bind to the substrate receptor and LC3-II interactive protein p62 to be escorted into autophagosomes for lysosomal degradation [16,17].

MAP1S (MAP1 small form), previously named C19ORF5 (chromosome 19 open reading frame 5), associates with microtubules stabilized by either chemotherapeutic drug and microtubule stabilizer taxanes or the tumour-suppressor protein RASSF1A (Ras-association domain family 1 isoform A) [18,19]. Specific accumulation of MAP1S short chain in responding to mitotic arrest leads to mitochondrial collapse on the mitotic spindle and mitotic cell death [20]. As a sequence homologue of MAP1A and MAP1B, MAP1S similarly interacts with mammalian autophagy marker LC3 [11,13,21], and bridges autophagy components with microtubules and mitochondria to affect autophagosomal biogenesis and degradation and suppress genome instability and tumorigenesis [13,22,23].

LRPPRC (leucine-rich pentatricopeptide repeat-containing), also known as LRP130, has been characterized as a mitochondrion-associated protein [24,25]. It was suggested that mutations in the gene cause LSFC (Leigh syndrome, French-Canadian type), a human disorder characterized by neurodegeneration and cytochrome *c* oxidase deficiency [26]. On the basis of the somatic mutation data of 17301 genes from 316 ovarian cancer patients from the Cancer Genome Atlas, mutations in *LRPPRC* were found to reduce the survival of patients [27]. Recently, it has also been reported that suppression of LRPPRC with LRPPRC-specific siRNAs causes the reduction in the infectivity of HIV-1 [28]. Previous studies continuously focused on its nucleic acid-associated functions such as transcriptional or translational regulation in nuclei, mitochondria or endoplasmic reticulum [29–39].

The confirmed interaction of LRPPRC with MAP1S [24,25,40] prompted us to investigate whether LRPPRC plays any roles in the regulation of autophagy and mitophagy. In the present study, we show that LRPPRC interacts with Beclin 1 and Bcl-2 and enhances the stability of Bcl-2. In such a way, more Beclin 1 is sequestered by Bcl-2 and prevented from association with PI3KCIII to initiate autophagy through the PI3K/Akt/mTOR pathway. Depletion of LRPPRC results in decreases in Bcl-2 levels and activation of basal levels of autophagy which enhances clean-up of dysfunctional mitochondria during the normal cell cycle. The LRPPRC depletion-induced autophagy activation occurs upstream of the conversion of LC3-I into LC3-II that is mediated by protein conjugate ATG5–ATG12 and is suppressed in cells with ATG5 depleted. LRPPRC therefore serves as a checkpoint protein for the initiation of basal levels of autophagy and enhances the removal of dysfunctional mitochondria and other cellular debris.

## MATERIALS AND METHODS

### Antibodies, siRNAs, plasmids and other reagents

Antibody against LRPPRC (1B8) [38,39] was a gift from Dr Serafín Piñol-Roma (Sophie Davis School of Biomedical Education, City College of New York, New York, NY, U.S.A.). Antibody against human LC3 (NB 100-2331) was purchased from Novus Biologicals. Antibodies against Bcl-2 (2870) and PI3KCIII (4263) were from Cell Signaling Technology. HRP (horseradish peroxidase)-conjugated secondary antibodies against mouse (172-1011) and rabbit (172-1019) were from Bio-Rad Laboratories. Antibody against Tom20 (translocase of the mitochondrial outer membrane 20) was from BD Transduction Laboratories (612278). Antibody against p62 was from Enzo Life Sciences International (BML-PW9860). Antibody against LAMP2 (lysosome-associated membrane protein 2) was from Abcam (ab37024). The IgG control antibodies from mouse (sc-2025) and rabbit (sc-2027), primary antibodies against β-actin (sc-47778), β-tubulin (sc-9104), cytochrome *c* (sc-7159), LRPPRC (mouse, sc-166178), ATG5 (sc-33210), LAMP1 (L1418), p27 (sc-528), Beclin 1 (sc-11427) and GFP (sc-8334), siRNA molecules specific to LRPPRC (sc-44734), p27 (sc-29429) and ATG5 (sc-41445), and random sequence control (sc-44234) were from Santa Cruz Biotechnology. FITC and rhodamine-conjugated secondary antibodies (A21206 and R6393), MitoTracker® Red CMXRos, Lipofectamine™ 2000 and Oligofectamine™ were from Invitrogen. GFP–LRPPRC carrying amino acids 139–1394, the end of human LRPPRC, was created as described in our earlier paper [24]. GFP–LC3 was supplied by Dr Mizushima [41]. Bafilomycin A1, NH_4_Cl and protease inhibitor cocktail were from Sigma. The Protein G–agarose beads, ECL Western blotting detection reagents and PVDF transfer membrane were from GE Healthcare.

### Cell transfection

Cell lines used for transfection included HeLa, HEK (human embryonic kidney)-293T or COS7 cells or HeLa cells stably expressing EGFP–LC3 (HeLa-GFP-LC3) that was established as described previously [5,41]. Lipofectamine™ 2000 was used to pack either siRNA molecules or plasmids following the manufacturer's recommended protocol. HeLa or HeLa-GFP-LC3 cells grown in six-well culture plates with or without coverslips to 30% confluence were transfected with random sequences or LRPPRC-specific siRNA for 72 h. Cells on coverslips were fixed for fluorescence microscopy and cells attached to the bottom of six-well plates for transmission electron microscopy analyses. Similarly, HeLa, HEK-293T, COS7 or HeLa-GFP-LC3 cells grown in six-well plates or 100-mm-diameter Petri dishes without coverslips were transfected with random sequences or LRPPRC, p27, ATG5-specific siRNA molecules individually or in combination for 48 or 72 h. Then, cells were harvested to prepare cell lysates directly for immunoblot (six-well plates) or for co-immunoprecipitation and then immunoblot analyses (100-mm-diameter Petri dishes). COS7 cells were transfected with either GFP control or GFP–LRPPRC plasmids for 24 h and harvested directly for immunoblot analyses. HeLa cells were first transfected with LRPPRC-specific siRNA. Cells were harvested 48 h later for immunoblot analyses.

### Co-immunoprecipitation assay

Lysates were prepared from HeLa or HEK-293T cells grown on 100-mm-diameter Petri dishes untreated, transfected with random or LRPPRC-specific siRNA. The cells were lysed in 1 ml of lysis buffer containing 150 mM NaCl, 1.0% Nonidet P40 and 50 mM Tris/HCl (pH 8.0) and total protein concentrations were quantified with the Pierce® BCA Protein Assay kit from Thermo Scientific. Same amounts of lysates with 1.6 mg of total proteins were subjected to immunoprecipitation with 2 μg of antibody against LRPPRC, Bcl-2, Beclin 1 or their respective IgG control antibody. The Protein G–agarose beads binding with antibodies and their associated proteins were precipitated and washed extensively with the lysis buffer five times. The final precipitates were resuspended in 100 μl of lysis buffer containing loading buffer and boiled for 5 min for immunoblot analyses.

### Immunoblot analyses

After boiled cell lysates were centrifuged, equal volumes of supernatants were loaded on polyacrylamide gels containing SDS and 10 or 15% (w/v) acrylamide depending on the molecular mass of the protein. Proteins were separated by electrophoresis, transferred on to PVDF membranes, bound with primary antibody and detected by ECL using HRP-conjugated secondary antibodies with ECL Western Blotting Detection Reagents. After exposure, X-ray films were developed, washed, dried and scanned into image files, and the relative intensity of a band to β-actin control were measured using ImageJ software (NIH).

### Fluorescence microscopy and transmission electron microscopy

HeLa and HeLa-GFP-LC3 cells untransfected or transfected with LRPPRC-specific siRNAs were treated with 20 mM NH_4_Cl for 12 h before fixation. Similarly to methods described in [5,20,24], cells were either incubated with 1 μM of MitoTracker® Red CMXRos in a 37°C cell culture hood for 30 min and then fixed with growth medium containing 3.7% formaldehyde for 15 min, or fixed directly with 4% (w/v) paraformaldehyde at 37°C for 30 min. After being permeabilized with 0.1% Triton X-100 for 20 min, cells were labelled with antibodies against LRPPRC, p62, Tom20, LAMP1, LAMP2 and/or cytochrome *c*, and their corresponding FITC or rhodamine-conjugated secondary antibodies. Images were captured with a laser-scanning microscope. The co-localization of LRPPRC with mitochondria was analysed by the plots of fluorescence intensities scanned along a line. The acquired images were converted into 8-bit binary files, and the total occupied area of GFP–LC3 punctate foci with a diameter greater than four pixels on each image were calculated using ImageJ software according to the manufacturer's manual.

### Transmission electron microscopy

HeLa cells transfected with LRPPRC-specific siRNA were treated with 10 nM bafilomycin A1 for 12 h. As we described previously for mouse tissue samples [13], cells were fixed for 1 h with a solution containing 3% (w/v) glutaraldehyde, 2% (w/v) paraformaldehyde and 0.1 M cacodylate buffer (pH 7.3). After fixation, the samples were washed and treated with 0.1% Millipore-filtered cacodylate-buffered tannic acid, post-fixed with 1% (w/v) buffered osmium tetroxide for 30 min, and stained *en bloc* with 1% (w/v) Millipore-filtered uranyl acetate. The samples were dehydrated in increasing concentrations of ethanol, infiltrated and embedded in LX-112 medium followed by polymerization in a 70°C oven for 2 days. Ultrathin sections were cut in a Leica Ultracut microtome (Leica), stained with uranyl acetate and lead citrate in a Leica EM Stainer, and examined in a JEM 1010 transmission electron microscope (JEOL) at an accelerating voltage of 80 kV. Digital images were obtained using the AMT Imaging System (Advanced Microscopy Techniques). Percentages of areas occupied by autophagic vacuoles or mitochondria were measured using the ImageJ program.

## RESULTS

### Suppression of LRPPRC leads to a decrease in mitochondrial potential

**Figure 1:**
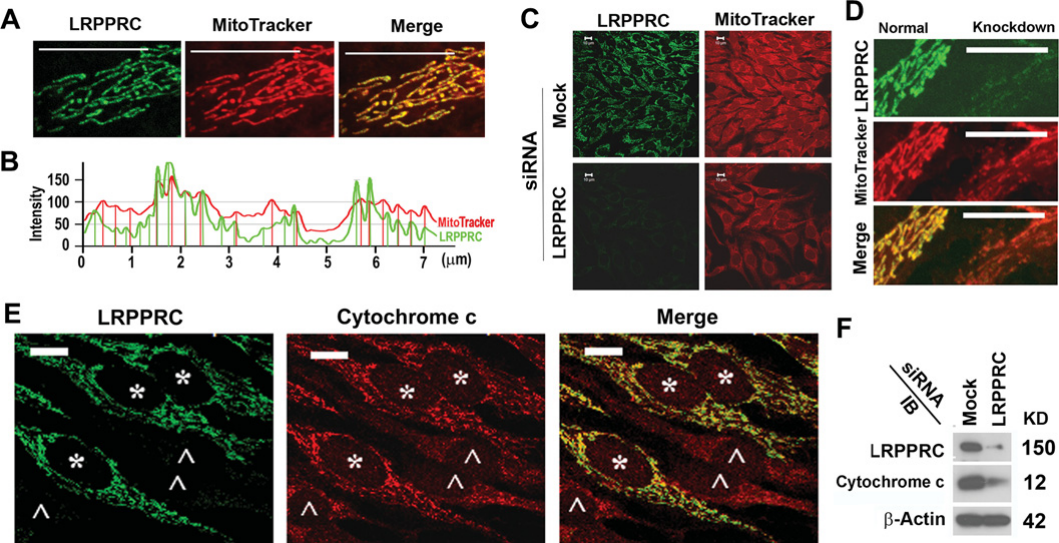
Depletion of LRPPRC leads to a decrease in mitochondrial potential (**A**) Immunostaining analysis showing the co-localization of antibody-stained LRPPRC (green) with MitoTracker-labelled mitochondria (red) in HeLa cells. (**B**) Plot of intensities of LRPPRC (green) and mitochondrial signals (red) presented in (**A**). (**C**) Analysis of the impact of LRPPRC depletion on the mitochondrial potentials labelled with MitoTracker (red) in HeLa cells treated with mock or LRPPRC siRNA for 72 h. LRPPRC were labelled with anti-LRPPRC antibody (green). (**D**) Comparison of MitoTracker intensities between two neighbouring cells with (right) and without (left) LRPPRC suppressed. (**E**) Comparison of cytochrome *c* signals (red) between cells retaining normal levels of LRPPRC (*, green) and cells with LRPPRC completely silenced (^∧^). (**F**) Immunoblot (IB) of lysates from HeLa cells treated without siRNA (Mock) or with siRNA specifically targeting LRPPRC (LRPPRC). Molecular masses are indicated in kDa. Scale bars in (**A**) and (**C**)–(**E**), 10 μm.

LRPPRC was originally reported as a member of hnRNP (heterogeneous nuclear ribonucleoprotein) complexes [38,39]. However, the endogenous LRPPRC protein was found to be generally associated with mitochondria (Figure 1A) as reported previously [24,26,38]. Examining the distribution in detail, we found that peaks of signals for LRPPRC and MitoTracker® Red CMXRos (referred to simply as MitoTracker hereinafter), a dye that labels mitochondria on the basis of their potentials, did not always overlap (Figure 1B). In general, LRPPRC distributes in specific sites of mitochondria, suggesting that some roles were unlikely to be associated with nuclear ribonucleoprotein complexes. We treated HeLa cells with a LRPPRC-specific siRNA to suppress the expression of LRPPRC and always achieved a high efficiency of suppression as confirmed by immunostaining or immunoblot analyses using an LRPPRC-specific monoclonal antibody (1B8) [38] (Figure 1). The LRPPRC-silenced cells exhibited dramatically reduced intensities of MitoTracker signals representing mitochondrial membrane potentials (Figure 1C). Furthermore, comparing two cells side by side, we found that the LRPPRC-silenced cells exhibited much weaker MitoTracker signals (Figure 1D). When mitochondria are depolarized, small proteins such as cytochrome *c* are released. Examining the cytochrome *c* content by immunofluorescence microscopy revealed that the cytochrome *c* intensities in LRPPRC-depleted cells were much weaker than those in cells with normal levels of LRPPRC (Figure 1E). Such a difference was confirmed further by immunoblotting the respective cell lysates (Figure 1F). Similarly, levels of cytochrome *c* in the majority of mitochondria with low LRPPRC levels were reported to be lower than in those with high LRPPRC levels in the fibroblasts from the same LSFC patient [36]. Unlike what was observed in acute apoptosis induction during which cytochrome *c* was released in the cytosol [42], the LRPPRC-depleted cells had lower levels of mitochondrial cytochrome *c* than the normal cells, but similarly undetectable levels of cytosolic cytochrome *c* as in normal cells. Consistent with the results from LRPPRC-knockout [29] and transgenic mice [37], LRPPRC-associated mitochondria retain higher levels of mitochondrial potentials.

### LRPPRC suppresses autophagy and its depletion leads to enhancement of autophagic flux

**Figure 2:**
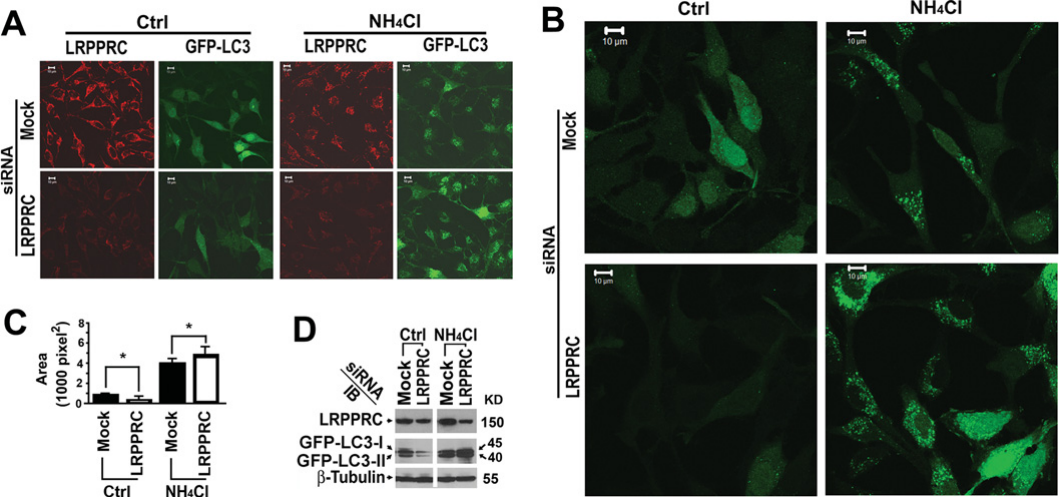
Suppression of LRPPRC leads to enhancement of basal levels of autophagy in HeLa cells stably expressing GFP–LC3 (**A**) Immunostaining of HeLa cells stably expressing GFP–LC3 treated with random siRNA (Mock) or LRPPRC-specific siRNA (LRPPRC) for 72 h in the absence (Ctrl) or presence of lysosomal inhibitor NH_4_Cl (20 mM overnight before harvest). (**B**) Enlarged views of GFP–LC3 punctate foci under similar treatments as shown in (**A**). (**C**) Quantification of GFP–LC3-labelled autophagosomes. The total area occupied by GFP–LC3 punctate foci is the mean±S.D. for ten randomly selected images in a field of 512 pixels×512 pixels. The significance of differences was determined by Student's *t* test. (**D**) Immunoblot (IB) of lysates from HeLa cells stably expressing GFP–LC3 similarly treated with random siRNA (Mock) or LRPPRC-specific siRNA (LRPPRC) in the absence (Ctrl) or presence of lysosomal inhibitor NH_4_Cl. Molecular masses are indicated in kDa.

Since LRPPRC was found to interact with MAP1S [24,25,40], a key regulator of the autophagy process [13], we were prompted to probe whether LRPPRC plays any role in autophagy. We used siRNA molecules to suppress the expression of LRPPRC in HeLa cells stably expressing GFP–LC3 (HeLa-GFP-LC3). A drastic reduction in GFP–LC3 fluorescence intensity was observed in cells with LRPPRC suppressed (Figure 2A). Such a reduction may suggest either inhibition of GFP–LC3 expression or acceleration of GFP–LC3 turnover. Although a portion of surface LC3-II is believed to be degraded before fusion of autophagosomes with lysosomes [2], inhibition of lysosomal activity with NH_4_Cl or bafilomycin A1 is expected to accumulate more GFP–LC3 punctate foci because basal levels of autophagy persist in different stages of the cell cycle [5]. The LRPPRC siRNA-treated HeLa-GFP-LC3 cells accumulated more GFP–LC3 punctate foci in the presence of NH_4_Cl than the untreated cells (Figures 2A–2C). To confirm the impact of LRPPRC suppression on basal levels of autophagy, we analysed the lysates of siRNA-treated HeLa-GFP-LC3 cells by immunoblotting. Consistent with the GFP–LC3 fluorescence intensities, the intensities of both GFP–LC3-I and GFP–LC3-II bands in the LRPPRC-depleted cells were reduced and then dramatically increased in the presence of NH_4_Cl (Figure 2D). Therefore LRPPRC suppression led to activation of GFP–LC3 turnover.

**Figure 3:**
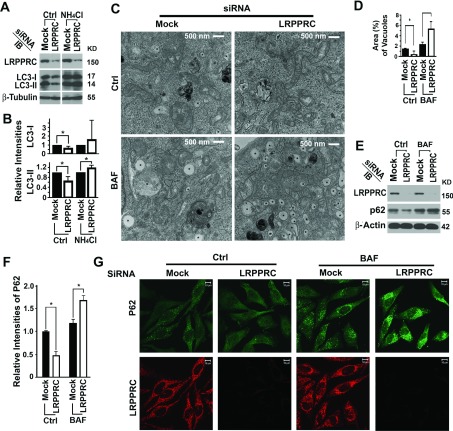
Suppression of LRPPRC leads to enhancement of basal levels of autophagy in native HeLa cells (**A**) Immunoblot analyses of LC3 isoforms in lysates from HeLa cells treated with random siRNA (Mock) or LRPPRC-specific siRNA (LRPPRC) in the absence (Ctrl) or presence of lysosomal inhibitor NH_4_Cl. Molecular masses are indicated in kDa. (**B**) Plots of relative intensities of LC3-I and LC3-II bands. The LC3-I and LC3-II intensities in samples treated with mock siRNA were set to 1. Results are means±S.D. of at least three repeats and the differences were compared using a paired Student's *t* test. *P≤0.05. (**C**) Transmission electron microscopy imaging of HeLa cells treated with random siRNA (Mock) or LRPPRC-specific siRNA (LRPPRC) in the absence (Ctrl) or presence (BAF) of lysosomal inhibitor bafilomycin A1. *, autophagy vacuoles. (**D**) Plot of percentages of area occupied by autophagy vacuoles in the transmission electron microscopy images. Results are means±S.D. of at least three repeats and the differences were compared using Student's *t* test. *P≤0.05. (**E**) Immunoblot (IB) analyses of p62 levels in lysates from HeLa cells treated with random siRNA (Mock) or LRPPRC-specific siRNA (LRPPRC) in the absence (Ctrl) or presence (BAF) of bafilomycin A1. (**F**) Plots of relative intensities of p62. The p62 intensities in samples treated with mock siRNA were set to 1. Results are means±S.D. of at least three repeats and the differences were compared using a paired Student's *t* test. *P≤0.05. (**G**) Immunostaining analysis of p62 levels in HeLa cells treated with random siRNA (Mock) or LRPPRC-specific siRNA (LRPPRC) in the absence (Ctrl) or presence (BAF) of bafilomycin A1.

To confirm that the suppressive role of LRPPRC on autophagy is not restricted to stable HeLa-GFP-LC3 cells, we performed LRPPRC suppression experiments in HeLa cells not expressing GFP–LC3. Similar trends were observed for endogenous LC3-I and LC3-II levels (Figures 3A and 3B). Examination of autophagic structures in cells in detail using transmission electron microscopy confirmed further the results from fluorescence microscopy and immunoblot analyses. The LRPPRC-suppressed cells contained less autophagic vacuoles in the absence, but more autophagic vacuoles in the presence, of the lysosomal inhibitor bafilomycin A1 (Figures 3C and 3D). p62/SQSTM1 (sequestosome 1) acts as a substrate receptor to bind with polyubiquitinated protein aggresomes and/or dysfunctional mitochondria and escort them to autophagosomes for degradation [17,43]. We confirmed further that levels of p62 were decreased upon LRPPRC suppression, but were increased in the presence of bafilomycin A1, similar to the levels of LC3 using immunochemical approaches (Figures 3E–3G).

**Figure 4:**
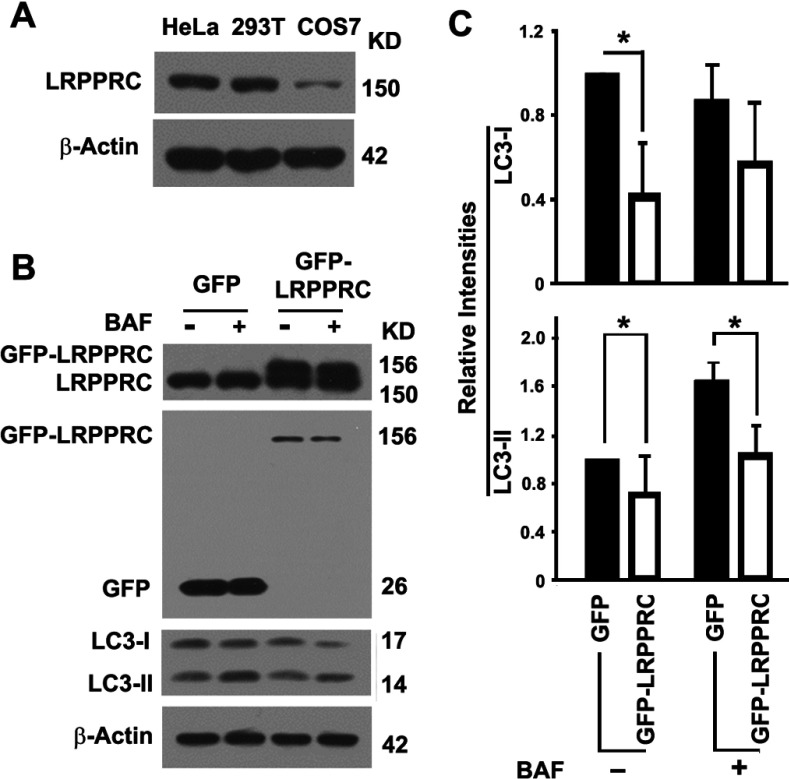
Overexpression of LRPPRC leads to inhibition of basal levels of autophagy in COS7 cells (**A**) Immunoblot analysis of LRPPRC protein levels in different types of cells. Lysates with the same amount of total proteins prepared from HeLa, HEK-293T and COS7 cells were compared. Molecular masses are indicated in kDa. (**B**) Immunoblot analyses of LC3 levels in lysates from COS7 cells overexpressing GFP or GFP–LRPPRC in the absence (−) or presence (+) of lysosomal inhibitor bafilomycin A1 (BAF). Expression levels of LRPPRC were confirmed by immunoblotting with antibodies against LRPPRC (top panel) or GFP (second panel). Molecular masses are indicated in kDa. (**C**) Plots of relative intensities of LC3-I and LC3-II bands with representative images shown in (**B**). The intensities in cells expressing GFP in the absence of lysosomal inhibitor were set to 1. Results are means±S.D. of at least three repeats and the differences were compared using a paired Student's *t* test. *P≤0.05.

Expression levels of LRPPRC in different cell lines were different: similar between HeLa and HEK-293T, but dramatically lower in COS7 cells (Figure 4A). Different cell lines may have different autophagy activities as a result of different dynamic balances among different autophagy regulatory components (results not shown). However, suppression of LRPPRC in HEK-293T cells exerted a similar impact on autophagy as in HeLa cells (results not shown). We selected COS7 cells to overexpress LRPPRC because of their lower levels of endogenous expression than HeLa and HEK-293T cells. The LRPPRC levels were readily doubled through overexpression (Figure 4B). Overexpression of LRPPRC in COS7 cells led to a decrease in levels of both LC3-I and LC3-II, suggesting that the two key steps controlling autophagy flux, the conversion of precursor LC3 into LC3-I and the conversion of LC3-I into LC3-II, may have been impaired (Figures 4B and 4C). LRPPRC suppression led to autophagy activation which resulted in the efficient conversion of LC3-I into LC3-II and degradation of LC3-II through lysosomes, whereas LRPPRC overexpression led to the formation of a weak autophagy flux. Thus LRPPRC acted as a checkpoint protein to suppress autophagy flux: the initiation of autophagy and the degradation of autophagosomes.

### Autophagy activation resulting from LRPPRC suppression leads to enhancement of mitochondrial turnover through mitophagy

**Figure 5:**
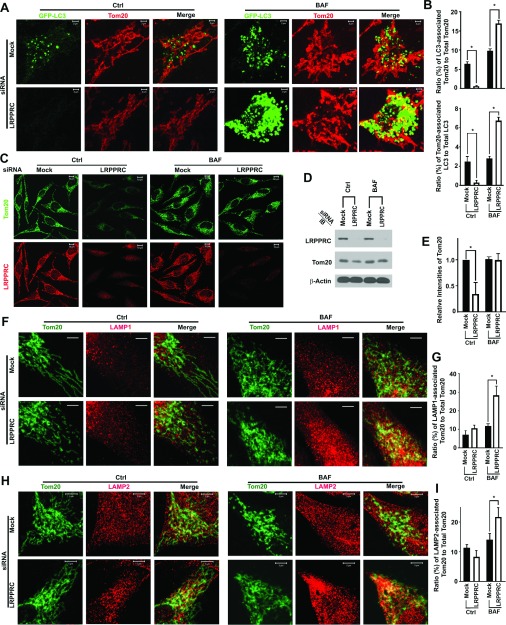
Suppression of LRPPRC leads to activation of mitophagy in HeLa cells (**A**) Fluorescence imaging analysis of the co-localization of GFP–LC3 with the mitochondrial marker Tom20. HeLa cells stably expressing GFP–LC3 treated with random siRNA (Mock) or LRPPRC siRNA (LRPPRC) for 72 h and with (BAF) or without (Ctrl) bafilomycin A1 in the last 12 h. (**B**) Plots of ratio (percentages) of GFP–LC3-associated Tom20 to total Tom20 (upper panel) or Tom20-associated GFP–LC3 to total GFP–LC3 (lower panel) with representative images shown in (**A**). Results are means±S.D. of at least three repeats and the differences were compared using Student's *t* test. *P≤0.05. (**C**) Immunostaining analysis of Tom20 levels in HeLa cells treated with random siRNA (Mock) or LRPPRC-specific siRNA (LRPPRC) in the absence (Ctrl) or presence (BAF) of bafilomycin A1. (**D**) Immunoblot (IB) analyses of Tom20 levels in lysates from HeLa cells treated with LRPPRC siRNA for 72 h and bafilomycin A1 (BAF) in the last 12 h. (**E**) Plots of relative intensities of Tom20 bands with representative images shown in (**D**). Results are means±S.D. of at least three repeats and the differences were compared using a paired Student's *t* test. *P≤0.05. (**F**) Fluorescence imaging analysis showing the co-localization of Tom20 with lysosomal marker LAMP1. HeLa cells treated with random siRNA (Mock) or LRPPRC siRNA (LRPPRC) for 72 h and with (BAF) or without (Ctrl) bafilomycin A1 in the last 12 h. Scale bars, 10 μm. (**G**) Plots of ratio (percentages) of LAMP1-associated Tom20 to total Tom20 with representative images shown in (**F**). Results are means±S.D. of at least three repeats and the differences were compared using Student's *t* test. *P≤0.05. (**H**) Fluorescence imaging analysis showing the co-localization of Tom20 with lysosomal marker LAMP2. HeLa cells treated with random siRNA (Mock) or LRPPRC siRNA (LRPPRC) for 72 h and with (BAF) or without (Ctrl) bafilomycin A1 in the last 12 h. (**I**) Plots of ratio (percentages) of LAMP2-associated Tom20 to total Tom20 with representative images shown in (**H**). Results are means±S.D. of at least three repeats and the differences were compared using Student's *t* test. *P≤0.05.

It was reported that autophagy is robust during different stages of the cell cycle to clean up damaged organelles such as mitochondria [5]. To examine the nature of the accumulated GFP–LC3 punctate foci, we stained cells with an antibody against Tom20 that is present in both functional and dysfunctional mitochondria [17]. We observed that few mitochondria labelled with Tom20 were co-localized with GFP–LC3 punctate foci upon LRPPRC suppression. Lysosomal blockade led to accumulation of GFP–LC3 punctate foci. However, a significantly greater number of mitochondria was co-localized with GFP–LC3 punctate foci in LRPPRC-deficient cells than in untreated cells (Figures 5A and 5B), which suggests that LRPPRC suppression enhanced mitophagy. To confirm that the activation of mitophagy resulted from LRPPRC suppression, we found that the levels of Tom20 in LRPPRC-suppressed cells were significantly lower than those in untreated cells (Figures 5C–5E). When mitophagy was blocked with the lysosomal inhibitor, higher percentages of Tom20-labelled mitochondria were co-localized with both lysosomal markers LAMP1 and LAMP2 in the LRPPRC-suppressed cells than in the untreated cells (Figures 5F–5I). Therefore the LRPPRC suppression enhanced turnover of mitochondria through autophagy and led to a reduction in mitochondrial mass.

### Autophagy activation resulting from LRPPRC depletion occurs upstream of the conversion of LC3-I to LC3-II

**Figure 6:**
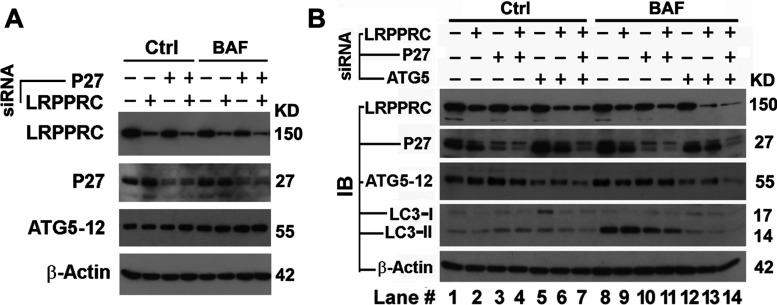
Activation of autophagy resulting from LRPPRC depletion occurs upstream of the conversion of LC3-I into LC3-II (**A**) Immunoblot analyses showing the impact of LRPPRC on the levels of p27 and ATG5. HeLa cells were treated with siRNA specific to LRPPRC or p27 either individually or in combination for 72 h in the absence (Ctrl) or presence (BAF) of bafilomycin A1 (10 nM overnight overnight before harvest). (**B**) Immunoblot analyses showing the impact of p27 and ATG5 on LRPPRC-suppressed autophagy initiation. HeLa cells were treated with siRNA specific to p27, ATG5 and/or LRPPRC individually or in combination for 72 h in the absence (Ctrl) or presence (BAF) of bafilomycin A1 (10 nM overnight overnight before harvest). Molecular masses are indicated in kDa.

To understand the mechanism by which LRPPRC depletion leads to autophagy activation, we investigated the impact of LRPPRC depletion on levels of proteins relating to autophagy initiation. Initiation of autophagy is basically regulated by the mTOR pathway [44]. Anti-apoptotic protein Bcl-2 inhibits autophagy initiation through sequestering Beclin 1 that activates autophagy through the PI3K/Akt/mTOR pathway [7]. In contrast, Bcl-2 can also activate autophagy through a Beclin-1-independent LKB1/AMPK/mTOR pathway [8,9] by increasing the levels of p27 protein that further increases levels of ATG5 [45]. MAP1S depletion was found to reduce levels of Bcl-2 as well as those of p27 and further inhibit basal levels of autophagy [13]. Suppression of LRPPRC led to an unstable variation in the levels of p27 protein. Both the LRPPRC suppression-induced variation in p27 levels and suppression of p27 levels using p27-specific siRNA did not exert a significant impact on ATG5–ATG12 levels (Figure 6A). These results suggested that the LRPPRC suppression-activated autophagy is less likely to be modulated by signals from the p27-controlled LKB1/AMPK/mTOR pathway [8,13].

To examine further the impact of p27 and ATG5 on autophagy activity in LRPPRC-depleted cells, we attempted to suppress the expression of LRPPRC, ATG5 and p27 individually or in combination using their respective siRNA molecules. Independent of LRPPRC levels, suppression of p27 impaired autophagy flux as expected (Figure 6B, lanes 10 and 11 compared with lanes 8 and 9), whereas suppression of ATG5 led to a greater impairment of autophagy flux (Figure 6B, lanes 12 and 13 compared with lanes 8 and 9). Suppression of both ATG5 and p27 at the same time further blocked autophagy in the LRPPRC-depleted cells (Figure 6B, lane 14). Therefore the LRPPRC suppression-induced activation of basal levels of autophagy requires the presence of ATG5 and occurs upstream of the ATG5–ATG12-catalysed LC3-I into LC3-II conversion.

### LRPPRC interacts with Beclin 1 and Bcl-2 and sustains Bcl-2 levels to suppress autophagy initiation through the PI3K/Akt/mTOR pathway

**Figure 7:**
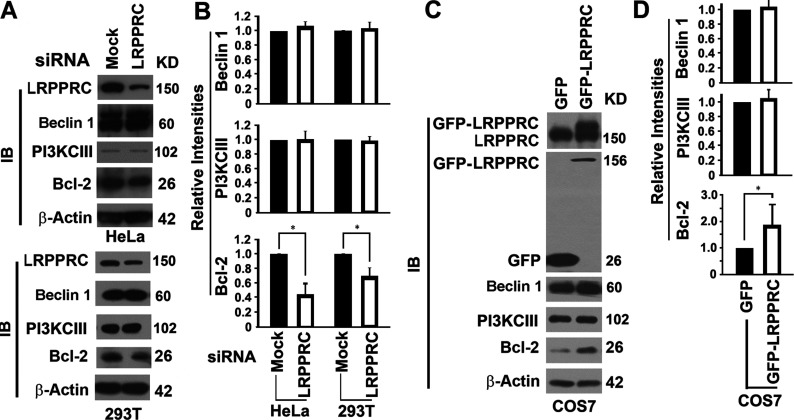
LRPPRC regulates the levels of Bcl-2 (**A**) Immunoblot (IB) analyses of lysates prepared from HeLa or HEK-293T cells treated with mock or LRPPRC siRNA for 72 h showing the impact of LRPPRC depletion on levels of proteins controlling autophagy initiation through the PI3K/Akt/mTOR pathway. Molecular masses are indicated in kDa. (**B**) Plots of relative intensities of Beclin 1, PI3KCIII and Bcl-2 bands as shown in (**A**). The intensities in samples treated with mock siRNA were set to 1. Results are means±S.D. of at least three repeats and the differences were compared using a paired Student's *t* test. *P≤0.05. (**C**) Immunoblot (IB) analyses of lysates prepared from COS7 cells overexpressing LRPPRC showing the impact of LRPPRC overexpression on levels of proteins controlling autophagy initiation through the PI3K/Akt/mTOR pathway. Expression levels of LRPPRC were confirmed by immunoblot with antibodies against LRPPRC (top panel) or GFP (second panel). Molecular masses are indicated in kDa. (**D**) Plots of relative intensities of Beclin 1, PI3KCIII and Bcl-2 bands in COS7 cells overexpressing LRPPRC as shown in (**C**). The intensities in cells overexpressing GFP were set to 1. Results are means±S.D. of at least three repeats and the differences were compared using a paired Student's *t* test. *P≤0.05.

In addition to regulation of autophagy through the p27-controlled LKB1/AMPK/mTOR pathway, Bcl-2 sequesters Beclin 1 so fewer Beclin 1 molecules bind to Vps34 (vacuolar protein sorting 34) PI3K (or PI3KCIII in mammalian cells) to form autophagy-inducing complexes [46]. To probe whether LRPPRC impacts autophagy initiation through the PI3K/Akt/mTOR pathway, we tested the impact of LRPPRC suppression on the levels of related proteins such as Bcl-2, Beclin 1 and PI3KCIII. Both Beclin 1 and PI3KCIII levels were not affected dramatically. Although Bcl-2 distributes in nuclear outer membrane, endoplasmic reticulum membrane and mitochondrial membranes [47], suppression of the exclusively mitochondria-associated LRPPRC alone led to a 60% or 35% reduction in Bcl-2 levels in HeLa or HEK-293T cells respectively (Figures 7A and 7B). Overexpression of LRPPRC in LRPPRC-deficient COS7 cells did not cause changes of either Beclin 1 or PI3KCIII levels, but did nearly double the amount of Bcl-2 protein (Figures 7C and 7D). Thus LRPPRC helps cells to sustain their levels of Bcl-2 protein.

**Figure 8:**
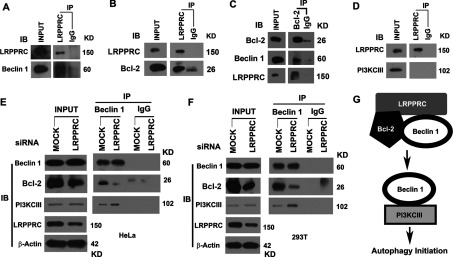
LRPPRC interacts with Beclin 1 and Bcl-2 and prevents Beclin 1 from forming an autophagy-inducing complex with PI3KCIII (**A**) Representative result of co-immunoprecipitation analyses of the LRPPRC–Beclin 1 interaction. The same amount of HeLa cell lysates was used to perform immunoprecipitation with the same amount of anti-LRPPRC antibody or mouse IgG control. (**B**) Representative result of co-immunoprecipitation analyses of the LRPPRC–Bcl-2 interaction. The same amount of HeLa cell lysates was used to perform immunoprecipitation with the same amount of anti-LRPPRC antibody or mouse IgG control. (**C**) Representative result of co-immunoprecipitation analyses of the interaction of Bcl-2 with LRPPRC and Beclin 1. The same amount of HeLa cell lysates was used to perform immunoprecipitation with the same amount of anti-Bcl-2 antibody or mouse IgG control. (**D**) Representative result of co-immunoprecipitation analyses of interaction of LRPPRC with PI3KCIII. The same amount of HeLa cell lysates were used to perform immunoprecipitation with the same amount of anti-LRPPRC antibody or mouse IgG control. (**E** and **F**) Co-immunoprecipitation analyses of the impact of LRPPRC depletion on Beclin 1–Bcl-2 and Beclin 1–PI3KCIII interactions. Lysates containing equal amounts of total proteins prepared from HeLa (**E**) or HEK-293T cells (**F**) treated with mock or LRPPRC siRNA were immunoprecipitated with anti-Beclin 1 or control IgG antibody and the precipitates were immunoblotted with antibodies against Beclin 1, Bcl-2 and PI3KCIII. (**A**)–(**F**) IB, immunoblot; IP, immunoprecipitation. Molecular masses are indicated in kDa. (**G**) Impact of LRPPRC depletion on the interaction of Beclin 1 with Bcl-2 or PI3KCIII.

To investigate the potential mechanism of how LRPPRC sustains Bcl-2 levels, we performed co-immunoprecipitation of endogenous proteins using specific antibodies. Both Beclin 1 and Bcl-2 were co-immunoprecipitated with LRPPRC (Figures 8A and 8B). In reverse, LRPPRC and Beclin 1 were also co-immunoprecipitated with Bcl-2 (Figure 8C). Beclin 1 was found to bind with PI3KCIII [46], whereas LRPPRC did not bind with PI3KCIII (Figure 8D). Therefore LRPPRC formed a complex with Bcl-2 and Beclin 1, but not with PI3KCIII.

We reasoned that LRPPRC forms a complex with Bcl-2 and Beclin 1 and prevents Beclin 1 from forming a complex with PI3KCIII to induce autophagy, and depletion of LRPPRC leads to degradation of Bcl-2 so that more Beclin 1 binds with PI3KCIII to initiate autophagy. We performed co-immunoprecipitation with Beclin 1-specific antibodies using lysates prepared from either HEK-293T or HeLa cells with either normal or suppressed levels of LRPPRC (Figure 8E and 8F). It was found that Beclin 1 protein in cells containing suppressed levels of LRPPRC bound with less Bcl-2, but more PI3KCIII (Figures 8E and 8G). Therefore LRPPRC suppression leads to enrichment of Beclin 1–PI3KCIII complexes and enhancement of autophagy.

## DISCUSSION

Initiation of autophagy is regulated either through the PI3K/Akt/mTOR pathway [7] or through the LKB1/AMPK/mTOR pathway [8,9]. The anti-apoptotic proteins of the Bcl-2 family exhibit opposite effects on autophagy initiation through the two different pathways. During the normal process of the cell cycle, mitochondria constantly generate energy to support cellular function and probably become damaged. Thus robust autophagy/mitophagy is sustained to remove those damaged mitochondria [5]. LRPPRC associates with mitochondria and interacts with Bcl-2 family proteins on mitochondria to maintain mitochondrial membrane potentials (Figure 1). Reduction in Bcl-2 levels results in release of Beclin 1 that will bind with PI3KCIII, the mammalian homologue of Vps34, to enhance initiation of autophagy [7,10,46] (Figure 8G). Similarly, suppression of LRPPRC with siRNA leads to a decrease in the levels of Bcl-2 protein, which results in the release of more Beclin 1 to bind with PI3KCIII and activate autophagy.

Autophagy initiation includes the nucleation and elongation of phagophore. Nucleation depends on Beclin 1–Vps34–Vps15 core complexes and other proteins, whereas elongation is closely associated with ATG5–ATG12 conjugate-mediated processing of LC3-I to LC3-II [48]. The LRPPRC depletion-induced autophagy activation occurs when nascent membranes are fused at their edges to form double-membraned autophagosomes. Although LRPPRC depletion does not exhibit any impact on the levels of p27 and ATG5, such activation of autophagy requires the p27- and ATG5-regulated elongation of the phagophore to be amplified. Therefore autophagy is suppressed when the elongation step is terminated by ATG5 suppression.

On the basis of bioinformatics analyses, LRPPRC was predicted to exhibit extended superhelical structures representing primitive structures that have been duplicated and evolved for diverse and specialized protein–protein recognition interfaces [49]. Similar to a lot of other proteins exhibiting extended superhelical structures, LRPPRC may simply serve as a scaffold protein to facilitate assembly of different functional protein complexes to affect multiple cellular processes. Previously, we have identified multiple interactive proteins including MAP1S, UXT (ubiquitously expressed transcript protein), haem-binding protein SOUL, CECR2 (cat eye syndrome chromosome region candidate 2) and fibronectin on the basis of only one of the multiple domains of LRPPRC [49]. The close relationship of LRPPRC with MAP1S in the regulation of organelle trafficking and mitophagy was suggested previously [20,22], but is in an extensive process of characterization. Whether the interaction with other proteins is autophagy-related is still under investigation. In the present paper, Bcl-2 and Beclin 1 have been added to the list of LRPPRC-interacting proteins, clearly suggesting a primary role of LRPPRC as a checkpoint protein for mitochondria turnover through autophagy. It will not be surprising if more interactive proteins of LRPPRC designate multiple diversified functions of LRPPRC in future.

Currently, it has been confirmed that LRPPRC is exclusively a mitochondrion-associated protein [24,26,36,38,50,51] (Figure 1). There is no hindrance in logic in the context of autophagy to explain any roles of LRPPRC in mitochondrion-associated processes such as post-transcriptional regulation of mitochondrial mRNA levels [29,34,52]. The close association of membrane structures of mitochondria with endoplasmic reticulum and nuclei may convince people that LRPPRC associates with nuclei or endoplasmic reticulum [53]. We believe that any association of LRPPRC with nuclear mRNAs is more likely to exist in cytosol where subcellular organelles containing nuclear mRNAs such as ribosomes are turned over through autophagy.

Most published results suggest that LRPPRC post-transcriptionally regulates gene expression in mitochondria [29]. All complexes involved in oxidative phosphorylation were down-regulated due to LRPPRC suppression in cultured cells [36], whereas only proteins in complex IV were specifically decreased due to LRPPRC depletion in knockout mice [29,34]. The generalized assembly defect in all oxidative phosphorylation complexes may be caused by the LRPPRC deficiency-enhanced mitophagy in cultured cells. However, the specific reduction in proteins in complex IV possibly resulted from defects in addition to mitophagy acceleration. It is known that LRPPRC interacts with eIF4E (eukaryotic initiation factor 4E) and selectively controls the nuclear export of several eIF4E-sensitive mRNAs [54]. LRPPRC forms an RNA-dependent protein complex to maintain a pool of non-translated mRNAs in mammalian mitochondria [29]. Therefore misregulated translation induced by LRPPRC deficiency potentially leads to the specific reduction in proteins in complex IV. Since 4E-BP1 (eIF4E-binding protein 1) acts as a switch between autophagy and protein synthesis [55], LRPPRC depletion may cause the conversion from protein translation into autophagy. The mRNA-associated ribosomes are selectively degraded by autophagy after being ubiquitinated [56,57], and a similar mechanism seems to be conserved in eukaryotic or mammalian cells [58,59]. It is therefore possible that differential removal of endoplasmic reticulum and endoplasmic reticulum-associated ribosomes through autophagy contributes to the specific reduction in proteins in complex IV.
